# Bill Regulating Patent Medicines

**Published:** 1904-10

**Authors:** 


### Bill Regulating Patent Medicines.

Massachusetts has a new bill providing for the control of the patent medicine business. It provides for the formula being printed on the label and provides for a fine of 50 cents for each package not so marked. The bill will be vigorously fought by the interests engaged in the patent medicine business. It is proposed to make the fine for the failure to label each package so high that it will make the payment, as a sort of tax, practically prohibitive. The full bill has not been given to the public.

